# Granulocyte Macrophage Colony Stimulating Factor Supplementation in Culture Media for Subfertile Women Undergoing Assisted Reproduction Technologies: A Systematic Review

**DOI:** 10.1155/2013/704967

**Published:** 2013-02-21

**Authors:** Charalampos Siristatidis, Paraskevi Vogiatzi, George Salamalekis, Maria Creatsa, Nikos Vrachnis, Demián Glujovsky, Zoe Iliodromiti, Charalampos Chrelias

**Affiliations:** ^1^Assisted Reproduction Unit, Third Department of Obstetrics and Gynecology, University of Athens, Rimini 1, Chaidari, 12642 Athens, Greece; ^2^Second Department of Obstetrics and Gynecology, Aretaieion Hospital, University of Athens, Vas Sofias 76, 11528 Athens, Greece; ^3^Médico Especialista en Medicina Reproductiva (SAMeR), Especialista Universitario en Ginecología y Obstetricia, Magíster en Efectividad Clínica, Viamonte 1432, Buenos Aires, Argentina; ^4^Third Department of Obstetrics and Gynecology, University of Athens, Attikon Hospital, Rimini 1, Chaidari, 12642 Athens, Greece

## Abstract

Granulocyte macrophage colony stimulating factor (GM-CSF) is a cytokine/growth factor produced by epithelial cells that exerts embryotrophic effects during the early stages of embryo development. We performed a systematic review, and six studies that were performed in humans undergoing assisted reproduction technologies (ART) were located. We wanted to evaluate if embryo culture media supplementation with GM-CSF could improve success rates. As the type of studies and the outcome parameters investigated were heterogeneous, we decided not to perform a meta-analysis. Most of them had a trend favoring the supplementation with GM-CSF, when outcomes were measured in terms of increased percentage of good-quality embryos reaching the blastocyst stage, improved hatching initiation and number of cells in the blastocyst, and reduction of cell death. However, no statistically significant differences were found in implantation and pregnancy rates in all apart from one large multicenter trial, which reported favorable outcomes, in terms of implantation and live birth rates. We propose properly conducted and adequately powered randomized controlled trials (RCTs) to further validate and extrapolate the current findings with the live birth rate to be the primary outcome measure.

## 1. Introduction

Assisted reproduction technologies (ARTs) are those methods that treat subfertility by providing the medical means to overcome the pathological obstacles in the fertilization process in a controlled laboratory environment, mimicking to a large extent the biological reproductive processes. Various factors have an impact on these processes, with the unfortunate upshot that successful fertilization does not invariably lead to the birth of a healthy offspring, as the growth and development of even confirmed embryo(s) in the laboratory does not always result in successful implantation, clinical pregnancy, and birth. For this reason, different culture media of the preimplantation embryo have been developed, which first appeared on the market in the early 80s [1]. Regrettably, robust evidence on their impact on pregnancy rates and embryo quality in women undergoing *in vitro* fertilization (IVF) cycles does not exist, while the relevant Cochrane review is still at the protocol stage [2].

Granulocyte macrophage colony stimulating factor (GM-CSF) is a cytokine/growth factor produced by epithelial cells under the influence of estrogens, in the human uterus and oviducts, which promotes hematopoiesis through monocyte and granulocyte proliferation and differentiation [3, 9]. Both the cytokine and the mRNA for its receptor are expressed and secreted by cells within the human ovary such that a main role for GM-CSF in the local regulation of ovarian events has been suggested, investigated, and discovered [10]. In reproduction, GM-CSF is highly involved in the embryo development process as it enhances embryonic growth and viability by exerting a positive control over various genetic paths, such as cell proliferation, progression to blastocyst, zona pellucida hatching, and embryo implantation in the endometrium, observed in humans [3] and animal studies [11]. Its incorporation into clinical research to establish whether its application could produce a positive outcome in an IVF setting has yielded encouraging results [3–8]. Its survival-promoting effects on the inner cell mass of embryos grown *in vitro* and *in vivo* characterize its embryotrophic properties [3, 12], which are essential for embryo development at early stages, shortly before and after its implantation. 

The aim of this study is to systematically review the published data on the benefit of GM-CSF with regard to outcome parameters in women undergoing ART.

## 2. Materials and Methods

This systematic review was conducted in accordance with the PRISMA guidelines [13] and in line with the a* priori* protocol approved by all authors. 

### 2.1. Search Strategy for the Identification of Studies

A broad range search strategy was developed using PubMed, with no language or study design restrictions and search period running from 1966 to August 2012. Reference lists of relevant articles were hand-searched for potentially eligible studies (“snowball” procedure). The National Institute of Clinical Excellence (NICE) fertility assessment and treatment guidelines were also hand-searched. Relevant “letters to the editor” and data coming from conferences on previously published or unpublished series were examined for potentially useable information.

Study authors were tried to be contacted for methodological clarifications, especially for missing data.

### 2.2. Study Eligibility

Studies assessing the effect of GM-CSF, employed as a medium supplement, on outcome parameters in human IVF programs, as compared with a matched control group where this intervention was not performed were considered in this systematic review. All study designs were included.

Case series, case reports, and animal studies were excluded. 

Collected data included general information (title, author, year, journal, and geographical and clinical settings), study characteristics (design, followup, and inclusions/exclusions), participants' characteristics (cause and duration of preexisting subfertility, age of the women, and different protocols for ovarian stimulation IVF, different culture media, conditions, and GM-CSF concentrations), and results (number of participants, reference population) as reported in Table 1.

In order to reduce bias (extraction, recording, conformity, and retrieval), three authors (CS, EV, and MC) performed the primary evaluation of titles and abstracts identified through the search and provided the list of potentially eligible studies. Each author extracted the data independently from the others, using the agreed data extraction form. If multiple publications using the same cohort were identified, the most recent or more complete publication was used for data extraction, but information from all relevant publications was used if required. Two authors (CS and EV) performed the final selection of the potential eligible studies of this review. Disagreements were resolved by team consensus. 

Based on extracted data, the quality of the included studies was evaluated using the Quality of Reporting of Meta-analyses (QUOROM) [14] and the nine-item Newcastle-Ottawa Quality Scale, notably a widely used tool for the quality assessment of observational/nonrandomized studies [15]. 

A search strategy was carried out based on the following MESH headings: “Granulocyte Macrophage Colony Stimulating Factor” OR “GM-CSF” AND “Reproductive Medicine” OR “Reproductive Techniques, Assisted” OR “ART” OR “*In Vitro* Fertilization” OR “IVF” OR “Intracytoplasmatic Sperm Injection” OR “ICSI.” 

### 2.3. Outcome Measures

The primary outcome measure was live birth per woman/couple. Additional outcome measures included clinical pregnancy per woman/couple (defined as evidence of a fetal heart on ultrasound at seven weeks), miscarriage rate (defined as the number of miscarriages divided by the number of clinical pregnancies), fertilization rate (rate of oocytes fertilized per oocytes retrieved), and laboratories parameters, such as progression of embryos to blastocyst stage, blastocyst performance and hatching, and chromosomal abnormalities of the embryos.

## 3. Results

The search algorithm yielded 153 records; of them, 112 were excluded as irrelevant on the basis of title and abstract. The full-text articles of the remaining 41 studies were obtained and assessed according to the eligibility criteria. Six studies were identified [3–8]; of these, four were randomized controlled trials (RCTs) and two were prospective observational studies; of these, two RCTs and one observational were published only as abstracts, whereas one RCT was presented in the 27th Annual Meeting of ESHRE in 2011 and is not published yet. The results are presented in Table 2.

Sjöblom et al. [3] examined the effect of the addition of GM-CSF on *in vitro* embryo development donated by couples undergoing IVF; they found increased percentage of embryos that reached the blastocyst stage (75.5 versus 30%, *P* < 0.001), improved hatching initiation (78 versus 47%, *P* < 0.001), and rise in the number of cells in the blastocyst (total, inner cell mass and trophectoderm cells, all *P* < 0.03) by approximately 35%—the effect appearing to be more profound in the inner cell mass—along with a reduction of the period in culture until its full formation. The authors proposed its supplementation in culture media for the production of implantation competent blastocysts, independently of the quality of the embryos and the culture media systems used.

The same study group [5] using thawed 2–4 cell embryos of women undergoing IVF/ICSI, reported in an abstract form, through the utilization of TUNEL labeling, that cell death was more frequent when GM-CSF was not added to the culture medium of blastocysts (mean number of apoptotic cells 5.6 ± 3.0 versus 2.8 ± 1.4, *P* < 0.05). The authors concluded that the incidence of cell death in the inner cell mass of the blastocyst was significantly higher in media without GM-CSF and that the role of GM-CSF addition in promoting cell viability in early embryo development was crucial. 

Kim et al. [4] examined the effect of GM-CSF in embryo culture media in human ART programs. The study group recorded a significantly improved clinical pregnancy rate in the cytokine-enhanced media when compared to controls (46.1% versus 30.8%, *P* < 0.05) in both IVF/ICSI cycles, with the difference being more evident when only IVF was used (66.7% versus 37.3%, *P* < 0.05). They emphasized the importance of the cytokine addition in embryo development and implantation and concluded that GM-CSF exerts a beneficial effect as a medium supplement.

The RCT by Shapiro et al. [6]—available only as abstract—investigated the effect of media supplementation with GM-CSF in terms of embryo progression from 2pn oocyte to blastocyst in ART programs and compared the study group with a control group without using the cytokine. Although there was an increased number of cleavage stage cells per embryo (6.1 versus 5.8, *P* = 0.047) and a 50% increase in the proportion of embryos that reached the expanded blastocyst stage (1.6 versus 1.1, *P* = 0.001), pregnancy and implantation rates were the same (47 versus 50% and 32 versus 25%, resp.) irrespectively of the addition of GM-CSF. The authors concluded that the GM-CSF lead to enhanced embryo growth throughout its preimplantation development.

The recent multicenter RCT conducted by Agerholm et al. [7] investigated the effect on the ploidy rate in donated human oocytes after the addition of GM-CSF in culture media. The time of the study was from fertilization to day 3, and authors evaluated morphologically the number of top-quality and normally developed embryos and analyzed chromosomes 13, 16, 18, 21, 22, *X* and *Y* through fluorescence *in-situ* hybridization. The comparison of the supplementation of the cytokine and the use of placebo showed no effect of the addition with regard to the above parameters.

The largest multicenter (*n* = 13) RCT conducted by ORIGIO a/s, a medtech company headquartered in Måløv, Denmark, and listed on the Oslo Stock Exchange, presented the results when using GM-CSF (EmbryoGen) in the 27th Annual Meeting of ESHRE in 2011 [8]; investigators reported a statistical significant improvement of 44% (24.5 versus 17%) of the ongoing implantation rate and a significant improvement of 28% both in terms of per embryo transferred (22.3 versus 17.4%, *P* = 0.02) and per transfer cycle (29.6 versus 23.1%, *P* = 0.03) in the live birth rate in women with previous miscarriages undergoing IVF, when the cytokine was added to the media of embryos. 

The average quality of all studies was median, with the multicenter RCT of ORIGIO a/s [8] to demonstrate the highest level.

## 4. Discussion

This is the first systematic review on the addition of GM-CSF to embryo culture media. It highlights the potential role of GM-CSF when added to the culture media of the preimplantation embryo during IVF. Different study groups have produced favorable results as concerning the role of the specific cytokine in promoting cell proliferation, viability, and integral progression through the early stages of embryo development. All groups were in agreement regarding the beneficial effect of cytokine enhancement on pre-implantation embryo development, albeit some differing results were reported as to its effect on the implantation and clinical pregnancy rate, whereas only one—large though—RCT ended up with positive results in terms of live birth rates. 

On one hand, these findings are important, as blastocyst formation is to a degree indicative of normal embryo development progression and provides a sounder basis, on which the outcome following embryo transfer is predictable. On the other, implantation and especially clinical pregnancy rates, were not significantly improved in all three of the six studies of the review, where it was taken into account [4, 6, 8]. Of note, in the larger—carrying the best quality of evidence—RCT [8], in terms of randomization and sample size, both implantation and live birth rates were improved by 44 and 28%, respectively; a fact that theoretically leaves little doubt on the value of the addition of the cytokine.

Most of the studies reported parameters at the blastocyst stage; culture at this stage provides a means for autoselecting the most developmentally competent embryos for transfer to patients as well as for achieving a better synchronization between the embryo stage and uterine development [16]. Thus, the transfer of blastocysts to the uterus may lead to higher implantation rates and help to reduce the number of multiple births resulting from IVF [3]. These two parameters are the main determinants of the enhanced live birth rates that are observed when embryo transfer is performed at blastocyst stage as compared to that at day 2 or 3 [17]. 

In terms of quality, the current review has a number of limitations, which mainly reflect the respective limitations of the individual studies. Although a plethora of records were originally retrieved during our search, we ended up with only six applied on humans that were included in this review. The exclusion criteria did not allow the inclusion of animal studies, which were numerous. In the same context, another fundamental problem was that missing information limited our ability to explore a relationship between different drugs, doses, or protocols used for IVF, type of subfertility, age group, and pregnancy occurrence (especially live birth), although our initial intention was to explore these parameters. Of note, we found one study where GM-CSF was administered directly to women with habitual abortions in their history, ending up with favourable results [18].

The beneficial nature of GM-CSF in terms of clinical pregnancy rates has yet to be resolved, although there is good evidence for women with >1 miscarriage in their personal history, since published data presents differing results and contradicting views on the cytokine effect beyond embryo transfer, all of which require further research for optimal elucidation. We propose additional properly conducted and adequately powered RCTs to further substantiate and extrapolate the current findings with the live birth rate to be the primary outcome measure. These should preferably treat infertile women with similar baseline characteristics and causes of subfertility as the reference group so as to provide clear insight into the effects of the addition of the factor on IVF. As stated above, various confounders must be taken into account, such as age of the woman, type of subfertility, doses and/or durations of protocol drugs, socioeconomic index, and parity. Moreover, the target of these studies should preferably involve special subgroups of subfertile women, such as women with unexplained subfertility and repeated implantation failures or recurrent miscarriage and those of an advanced age. Finally, cost-effectiveness needs to be clearly demonstrated as well as, most importantly, any potential genetic and/or chromosomal consequences incurred by the supplementation of GM-CSF manifesting as abnormalities in the health of the individual in childhood and/or in adult life. It may be—unavoidably—some time before epidemiological studies and their proper systematic reviews and meta-analyses are able to compile assemble the follow-up times required to fully address long-term effects.

## Figures and Tables

**Table 1:** Study design parameters and characteristics.

Author, year	Study design	Number of patients	Patient characteristics and infertility factor	GM-CSF concentration	Control medium	ART
Sjöblom et al., 1999 [3]	Prospective Observational Australia	99 (2–4 cell embryos)	N/A	2 ng/mL	20 *μ*L microdrops IVF-50/S2 with insulin/G1.2 and G2.2 with reduced glutamine, EDTA and phosphate	IVF GnRH agonist, 150–225 IU/day r-FSH 10.000 IU hCG

Kim et al., 2001 [4]	Prospective Observational South Korea	154 (patients)	Women aged 28–39.6 yrs	2 ng/mL	10 *μ*L microdrops of modified P-1	IVF/ICSI

Sjöblom et al., 2001 [5]	RCT Australia	62 (2–4 cell embryos)	N/A	2 ng/mL	20 *μ*L drops of Sydney IVF Cleavage/1 mL Blastocyst Cook IVF	IVF/ICSI

Shapiro et al., 2003 [6]	RCT USA	72 patients with ≥2 pn oocytes	N/A	N/A	Quinn's Advantage Protein Plus	IVF

Agerholm et al., 2010 [7]	RCT Denmark	73 women donating 86 oocytes	Women aged 25–37 yrs Male factor: *n* = 53 Tubal factor: *n* = 17 Endometriosis: *n* = 5 PCOS/endocrine: *n* = 1 Unexplained: *n* = 18 Tubal insemination using donor Spermatozoa with proven fertility and normal karyotype	2 ng/mL	Blast Assist System Medium 1	IVF/ICSI Long protocols (GnRH agonists) and short protocols (GnRH antagonists)

Origio website, 2011 [8]	RCT 11 Danish 3 Swedish centers	1319 included 1322 enrolled (patients)	Women with >1 miscarriage 21–35 d cycle aged 25–39 years IVF/ICSI indications No oocyte donation	2 ng/mL	Media without GM-CSF	IVF/ICSI standard agonist/antagonist FSH 100–300 IU

**Table 2:** Outcome parameters: GM-CSF versus control.

	Embryo cell number (72 hrs)	Normal embryo development on day 3	Embryos reaching blastocyst stage	Hatching initiation of blastocysts	Total cell number in blastocysts (day 5)	Mitotic index	Mean number of apoptotic cells in blastocyst/TUNEL clusters	Embryos reaching morula stage	Clinical pregnancy rate	Chromosomal constitution of embryos	Implantation rate	Live birth rate
Sjöblom et al., 1999 [3]			76% versus 30%	78% versus 47%	35% higher with GM-CSF			80% increase in GM-CSF treatment				
Kim et al., 2001 [4]									35% versus 24%			
Sjöblom et al., 2001 [5]						3.5 ± 1.7 versus 3.3 ± 1.3	2.8 ± 1.4 versus 5.6 ± 3.0					
Shapiro et al., 2003 [6]	6.1 versus 5.8		50% increase in GM-CSF group						47% versus 50%		32% versus 25%	
Agerholm et al., 2010 [7]		50% versus 28.1%								34.8% versus 33.3%		
Origio website, 2011 [8]											24.5% versus 17%	29.6% versus 23.1% (per cycle) 22.3% versus 17.4% (per embryo transfer)
